# A Rare Occurrence of Spontaneous Coronary Artery Dissection in Elderly: Diagnostic Challenges and Clinical Implications

**DOI:** 10.7759/cureus.57464

**Published:** 2024-04-02

**Authors:** Ahmad W Haddad, Wassim Abouzeid, Noreen Mirza, Dilesha Kumanayaka, Eyad Ahmed, Joaquim Correia, Addi Suleiman

**Affiliations:** 1 Internal Medicine, Saint Michael's Medical Center, Newark, USA; 2 Cardiology, Saint Michael's Medical Center, Newark, USA

**Keywords:** reduced ejection fraction, heart failure, acute coronary syndrome, takosubo cardiomyopathy, cardiomyopathy, coronary artery stenosis

## Abstract

Spontaneous coronary artery dissection (SCAD) is the ripping of the epicardial coronary artery wall without any trauma, coronary procedures, or rupture of atherosclerotic plaque. Intimal rip, intramural hematoma, and false lumen formation are the hallmarks of this disease, which may result in coronary blood flow obstruction and myocardial ischemia. The role of SCAD in acute coronary syndrome (ACS), and sudden death has come to light more and more, particularly in young females and those with few typical atherosclerotic risk factors. This study details a 65-year-old female with a history of hypertension, hyperlipidemia, asthma, and chronic kidney disease who presented with severe chest pain and elevated troponin levels. Upon investigation, spontaneous dissection of the left anterior descending artery (LAD) involving its mid and distal segments was identified. The present case highlights a rare occurrence of spontaneous coronary artery dissection (SCAD) in a demographic typically unaffected by the condition - females aged 65 years and over. The atypical presentation underscores the importance of reporting such cases to prevent oversight. This patient's case is particularly noteworthy as it deviates from the typical predisposing factors associated with SCAD, such as youth, pregnancy, or stressors. Additionally, the case is unique in that it presented both SCAD and imaging findings consistent with takotsubo cardiomyopathy, suggesting a complex cardiac pathology deserving of further study and consideration.

## Introduction

Spontaneous coronary artery dissection (SCAD) is a rare and potentially life-threatening condition, the precise cause of SCAD remains unknown, it is often associated with young females and precipitating factors such as pregnancy or extreme emotional or physical stress, fibromuscular dysplasia, hormonal exposure [1,2]. Diagnosis typically involves acute coronary syndrome (ACS) presentation, ECG changes, elevated troponin levels, and echocardiogram abnormalities with the association of cardiac catheterization and angiography [3]. Follow-up includes computed tomography angiography (CCTA) and magnetic resonance angiography. Conservative management is favored initially, with most cases showing angiographic healing within a month. However, vigilance is crucial due to the risk of recurrence [3]. Angiography and CCTA can be used for diagnosis. Percutaneous coronary intervention (PCI) is reserved for cases with ongoing ischemia, with coronary artery bypass grafting (CABG) considered for certain scenarios [4].

## Case presentation

A 65-year-old female with a past medical history of tobacco abuse, hypertension, hyperlipidemia, asthma, medication non-compliance, and chronic kidney disease presented with chest pain started a day before presentation. The chest pain was located on her left side and started at rest. It was substernal and radiated to her left arm. Upon arrival, initial vital signs included a temperature of 98.1° Fahrenheit, heart rate of 76 beats per minute, respiratory rate of 12 breaths per minute, and oxygen saturation of 99% on room air. Physical examination was normal including cardiac examination with normal S1/S2, no S3, or S4 was present and there were no murmurs, rubs, or gallops.

**Figure 1:**
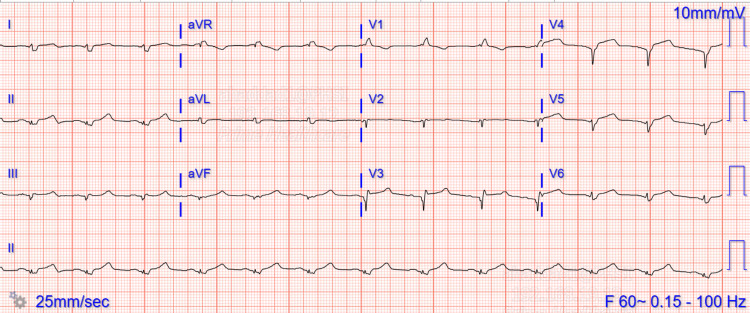
ECG of the patient at presentation. Sinus rhythm with borderline left axis deviation and ST segment elevations in the anterolateral leads.

**Figure 2:**
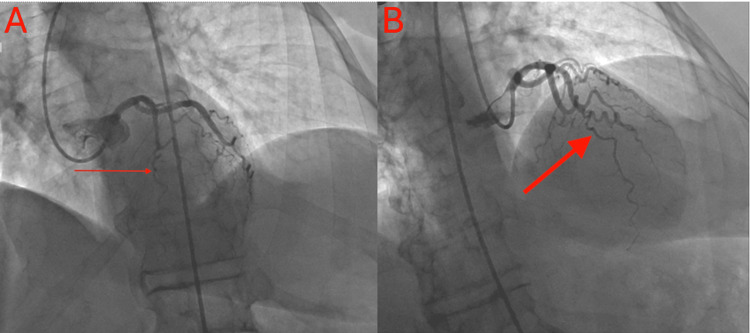
Different views of left heart angiogram. (A) Patent proximal LAD with narrowing and tapering lesion extending from the mid to distal lesions. (B) Narrowing and tapering lesion extending from mid to distal lesions.

**Figure 3:**
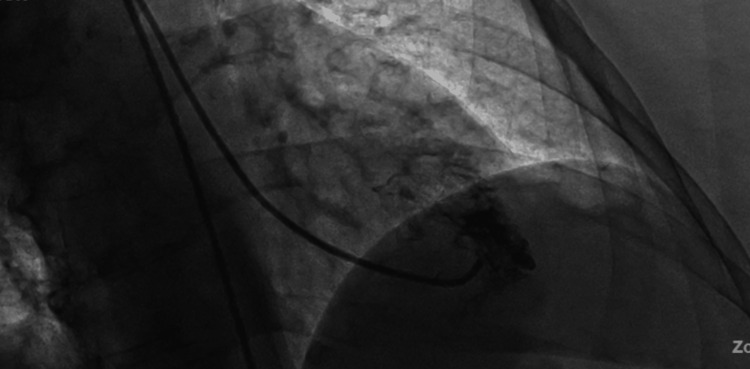
Left ventriculogram revealed apical ballooning and hypokinesis consistent with takotsubo cardiomyopathy.

Initial laboratory findings were significant for white blood cell count of 12.60×10^3^/μL, hemoglobin of 12.0 g/dL, potassium of 7.1 mmol/L, creatinine of 1.68 mg/dL and significantly elevated high sensitivity troponins at 11,184 ng/L which peaked at 21,517 ng/L. Electrocardiogram revealed normal sinus rhythm with borderline left axis deviation and ST segment elevations in the anterolateral leads (Figure 1). The patient was started on aspirin, ticagrelor, and therapeutic heparin drip. The patient was taken for emergent cardiac catheterization which was significant for mid to distal tapering of the left anterior descending artery (Figures 2A, 2B), and left ventriculogram revealed apical ballooning and hypokinesis consistent with takotsubo cardiomyopathy (Figure 3). The patient was continued only on aspirin and atorvastatin.

**Figure 4:**
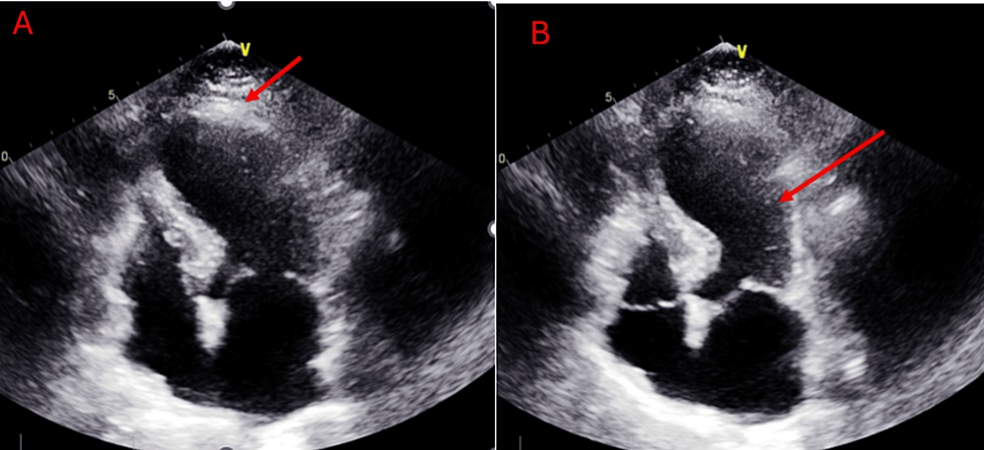
Apical four-chamber view of the heart. (A) Arrow pointing at the apical side of the heart shows signs of apical ballooning and akinesia and (B) arrow pointing at the left chamber of the heart shows ballooning of the left ventricle and a "Jar shape."

The transthoracic echocardiogram showed an ejection fraction of 35-40%. There was akinesis noted in the mid to apical septal walls, as well as in the entire apex, and partially in the mid anterior and lateral walls (Figures 4A, 4B). The patient was started on guideline-directed medical therapy for systolic heart failure including metoprolol succinate 25 mg daily and Entresto 24-26 mg twice daily, with aspirin 81 mg for primary prevention. Patient's troponin and chest pain went away on the day before admission, and she was instructed to stop smoking. Her follow-up appointment was scheduled for one week later. At that time, the patient denied any chest pain, shortness of breath, or leg swelling. ECG and troponin tests were conducted for monitoring. The ECG showed no T-wave elevations and troponin levels trended down to 215 ng/L.

## Discussion

The actual incidence of SCAD is unknown. For the next eight decades, following Pretty's initial case report of 1931, the majority of the data was derived from single case reports and tiny patient series [5]. A well-known research series found that among patients referred for coronary angiography, the prevalence ranged from 0.07% to 1.1%. [6-8]. Females are mostly affected by SCAD, the percentage of females in three of the major SCAD series ranges from 81% to 96%, with a mean age of 45-52 years at the time of the index event diagnosis [9].

Two theories have been put forth to explain how the false lumen in SCAD develops. According to the "inside-out" theory, an intramural hematoma (IMH) forms when the endothelial-intimal layer is disrupted, allowing blood from the lumen to enter the artery wall. According to the "outside-in" theory, an IMH without intimal disruption is produced by a bleeding episode that occurs within the coronary artery wall at the level of the vasa vasorum. Myocardial ischemia can occur in the vicinity of the afflicted coronary artery as a result of either mechanism, which can also cause hematoma extension and compression of the actual lumen [10].

A long list of conditions has been related to SCAD, either as predisposing factors that make a coronary artery wall structure more prone to dissection or as factors that precipitate acute episodes of SCAD, fibromuscular dysplasia, collagen vascular disorders, chronic inflammatory systemic diseases as systemic lupus erythematosus, inflammatory bowel disease, sarcoidosis, hypothyroidism, and pregnancy [11]. Several factors have been linked to the acute onset of SCAD. It was found that emotional stressors than physical stressors are the most dominant precipitating factors in up to 65% of the cases [12]. One interesting case was reported recently for a post-coital SCAD [13].

There is significant variation across different series in the percentage of individuals with SCAD that present as ST-segment elevation MI as opposed to non-ST-segment elevation MI (26-49%) [12,14]. Other clinical manifestations, including ventricular fibrillation or tachycardia, are rare (4-10%) [12]. In the Italian cohort, 3% of cases presented as out-of-hospital sudden cardiac death [14]. Ninety-six percent of MI patients in the Canadian cohort report presenting with chest pain, and half also report having discomfort radiating to the left upper limb. These early symptoms are typical of myocardial infarction (MI) patients. Notable symptoms were nausea/vomiting (24%) and sweating (21%), among others [15].

The primary method for diagnosing SCAD is currently invasive coronary angiography [16,17]. When intracoronary images became available, it was discovered that most patients of SCAD do not exhibit a double lumen pattern on angiography. Due to this discovery, SCAD was categorized using angiographic patterns that were distinct from those of iatrogenic dissections brought on by balloon angioplasty [18]. There are three major angiographic patterns in his classification. A double-lumen image is a characteristic of type 1 lesions. A lumen narrowing characterizes type 2 lesions, which often have a lesion length of more than 20 mm. Type 2 lesions are divided into two following subtypes: type 2b, which occurs when the IMH spreads distally to the end of the coronary artery, and type 2a, which occurs when the distal vessel regains its normal size.

Last but not least, type 3 lesions resemble atherosclerotic lesions in that they are characterized by an abrupt lumen narrowing with distal artery size recovery that restricts a focal lesion (length <20 mm) [11]. In general, compared to atherosclerotic disease, it typically affects more distant portions. Moreover, the left anterior descending coronary artery is the most commonly implicated artery. Compared to controls without coronary artery disease, patients with SCAD had more twisted blood arteries [19]. Other methods to diagnose are coronary CT angiography, optical coherence tomography, and intravascular ultrasound [11].

The course of treatment that was commonly used for SCAD was basically the same as what was advised for ACS brought on by atherosclerotic disease. Given the previously reported underlying physiopathology (which essentially involves a first bleeding episode that results in an IMH and a weakened coronary artery wall), it is unclear why strong antiplatelet therapy and lipid-lowering medications are necessary. However, some hypothesis-generating observational data has revealed that certain drugs might alter the likelihood of recurrences in those who have survived SCAD [11]. The majority of the information regarding the safety and effectiveness of thrombolysis in the setting of SCAD is derived from solitary case reports, which can include anything from favorable outcomes to coronary rupture and dissection extension [11]. There is ongoing debate on the usage of antiplatelet medicines and the length of therapy. In certain cases of SCAD, there may be an accompanying thrombus inside the actual lumen, which provides support for the addition of dual antiplatelet medication [11]. There needs to be more information regarding anticoagulant therapy while treating SCAD. If there is no other justification for anticoagulant medication and the patient has ACS, which is a common presentation of SCAD, anticoagulant therapy with heparin or fondaparinux should be stopped as soon as the diagnosis of SCAD is confirmed [17].

## Conclusions

This case emphasizes the importance of considering SCAD as a differential diagnosis, even if there are no traditional risk factors present, especially in the elderly population. It also highlights the diagnostic complexity when there are overlapping features of other cardiac conditions, such as takotsubo cardiomyopathy. SCAD is more commonly seen in young females and is rare in females aged 65 years or older. Reporting cases in older females is essential to avoid overlooking potential diagnoses. Our patient's case was atypical since it lacked typical SCAD risk factors such as youth, pregnancy, and stress. Moreover, it presented a unique combination of SCAD alongside imaging consistent with takotsubo cardiomyopathy. Since this patient also does not exhibit considerable stress that could cause takotsubo cardiomyopathy, the results of the angiography support the diagnosis of SCAD.
